# Risk factors and early warning scoring system for chronic postsurgical pain following video-assisted thoracoscopic surgery in elderly lung cancer patients: a retrospective cohort study

**DOI:** 10.3389/fgene.2026.1879530

**Published:** 2026-08-05

**Authors:** Degui Wang, Weichen Pan, Qinghua Shi, Yuhong Liu

**Affiliations:** 1 Department of Chest Surgery, Anhui Chest Hospital, Hefei, Anhui, China; 2 Department of Anesthesiology, Huai’an Clinical Medical College of Jiangsu University, Huai’an Hospital of Huai’an City, Huai’an, Jiangsu, China; 3 Department of Warehouse, Jinling Hospital, Medical School of Nanjing University, Nanjing, Jiangsu, China; 4 Department of Surgical Outpatient Dressing Change Center, Xianyang Hospital of Yan’an University, Xianyang, Shaanxi, China

**Keywords:** chronic postsurgical pain, early warning system, elderly patients, lung cancer, predictive model, risk factors, video-assisted thoracoscopic surgery

## Abstract

**Background:**

Chronic postsurgical pain (CPSP) is a clinically significant and frequently underrecognized complication of video-assisted thoracoscopic surgery (VATS) for lung cancer, affecting a substantial proportion of elderly patients and adversely impacting quality of life and long-term recovery. In elderly populations, the risk of CPSP is compounded by age-related physiological vulnerabilities, high comorbidity burden, and psychosocial factors that predispose to central sensitization and pain chronification. Despite growing awareness of CPSP as a distinct clinical entity under the IASP ICD-11 classification, validated risk stratification tools specific to elderly patients undergoing VATS remain lacking. This study aims to identify independent clinical risk factors for CPSP following VATS in elderly lung cancer patients and to develop a practical, evidence-based early warning scoring system to support preoperative risk stratification and targeted preventive interventions.

**Methods:**

A retrospective cohort study was conducted on 200 elderly patients (aged ≥65 years) who underwent VATS for lung cancer from 12 November 2023 to 13 August 2025 at a tertiary care center and were categorized into the CPSP group (n = 100) and non-CPSP group (n = 100) based on pain persistence beyond 3 months postoperatively. Comprehensive demographic, clinical, surgical, and perioperative data were collected. Univariate and multivariate logistic regression analyses were performed to identify independent risk factors. A predictive scoring system was developed based on regression coefficients, and its discriminatory performance was assessed using receiver operating characteristic (ROC) curve analysis.

**Results:**

The overall incidence of CPSP was 50.0% (100/200) in this elderly cohort. Multivariate logistic regression identified seven independent risk factors: advanced age (≥75 years, OR = 3.24, 95% CI: 1.78–5.89, p < 0.001), female gender (OR = 2.16, 95% CI: 1.24–3.76, p = 0.007), preoperative chronic pain (OR = 4.52, 95% CI: 2.15–9.51, p < 0.001), anxiety/depression (OR = 2.87, 95% CI: 1.52–5.42, p = 0.001), extensive lymph node dissection (OR = 3.08, 95% CI: 1.65–5.75, p < 0.001), intraoperative blood loss >50 mL (OR = 2.45, 95% CI: 1.32–4.54, p = 0.005), and inadequate acute postoperative pain control (OR = 3.76, 95% CI: 1.98–7.14, p < 0.001). A 0–17 point early warning scoring system was established: low risk (0–4 points, CPSP probability 8.3%), moderate risk (5–8 points, 47.7%), and high risk (≥9 points, 84.4%). The scoring system demonstrated good discriminatory performance with AUC of 0.847 (95% CI: 0.794–0.900), sensitivity 82.0%, and specificity 78.0% at the optimal cut-off of 7 points, pending external validation.

**Conclusion:**

This study identified seven independent risk factors for CPSP following VATS in elderly lung cancer patients and established a practical early warning scoring system with good predictive performance (AUC = 0.847), enabling preoperative risk stratification and facilitating targeted preventive interventions to improve long-term outcomes. External multicenter validation is warranted before clinical implementation.

## Introduction

Lung cancer remains the leading cause of cancer-related mortality worldwide, with the majority of cases diagnosed in elderly patients aged 65 years or older (Bray et al., 2018). Video-assisted thoracoscopic surgery (VATS) has become the standard surgical approach for early-stage non-small cell lung cancer, offering reduced surgical trauma, faster recovery, and shorter hospital stay compared to open thoracotomy (Bendixen et al., 2016). Despite these advantages, VATS is not without its long-term complications. Chronic postsurgical pain (CPSP), defined under the International Association for the Study of Pain (IASP) ICD-11 taxonomy as pain persisting at least 3 months after surgery in the operative area and not attributable to other causes (Macrae, 2008; Werner and Kongsgaard, 2014; Schug et al., 2019), represents one of the most clinically significant and underappreciated sequelae of thoracic surgery (Wildgaard et al., 2009). In elderly patients, the impact of CPSP is particularly profound given their reduced functional reserve, greater comorbidity burden, and heightened vulnerability to pain-related deterioration in quality of life.

The elderly surgical population is particularly susceptible to CPSP due to age-related neurobiological changes including impaired endogenous pain modulation, increased peripheral sensitization, and altered descending inhibitory control (Gagliese and Melzack, 1997; Gibson and Farrell, 2004). Comorbid conditions such as pre-existing chronic pain, anxiety, and depression further compound this risk by predisposing patients to central sensitization and pain chronification following nociceptive surgical stimuli (Kehlet et al., 2006; Katz and Seltzer, 2009; Chapman and Vierck, 2017; Glare et al., 2019). In addition, procedure-specific factors including the extent of lymph node dissection, degree of intraoperative tissue trauma, and adequacy of acute postoperative pain control areHudnall increasingly recognized as modifiable determinants of CPSP (Gerbershagen et al., 2014; Pogatzki-Zahn et al., 2017; Steinthorsdottir et al., 2014). Despite growing appreciation of the multifactorial nature of CPSP, systematic risk stratification tools specifically designed and validated for elderly patients undergoing VATS for lung cancer remain lacking.

CPSP following VATS carries substantial clinical and socioeconomic consequences. It impairs physical function, limits rehabilitation, disrupts sleep, and contributes to prolonged opioid use and healthcare utilization (Clarke et al., 2014; Correll, 2017). In elderly lung cancer survivors, persistent postoperative pain may accelerate functional decline, increase fall risk, and reduce adherence to adjuvant oncological therapies. Published incidence rates of CPSP following VATS range from 25% to over 50%, with the variability attributable to differences in patient populations, pain assessment methods, and follow-up durations (Bayman and Brennan, 2014; Wildgaard et al., 2016). The inconsistency in reported rates, combined with the absence of validated preoperative risk models tailored to elderly patients, underscores the urgent need for evidence-based tools to identify high-risk individuals before surgery.

Existing predictive models for CPSP have predominantly been derived from thoracotomy cohorts or mixed surgical populations, with limited applicability to the VATS-specific elderly lung cancer setting (Althaus et al., 2012; VanDenKerkhof et al., 2013; Meretoja et al., 2017). Moreover, validated scoring systems that integrate both patient-level risk factors (demographic, psychological, and comorbidity-related) and procedure-level variables (surgical complexity, intraoperative parameters, and acute pain control) in a single, clinically actionable framework are currently unavailable for this population. Development of such a tool would enable risk-stratified perioperative planning, targeted allocation of multimodal analgesia and psychological support, and early identification of patients at highest risk for pain chronification (Humble et al., 2015).

This study aims to identify independent clinical risk factors for CPSP following VATS in elderly lung cancer patients and to develop a practical, validated early warning scoring system for preoperative risk stratification. By integrating comprehensive demographic, clinical, surgical, and perioperative variables, we seek to provide clinicians with an actionable bedside tool that supports individualized perioperative planning, facilitates targeted preventive interventions, and ultimately improves long-term pain outcomes and quality of life in this vulnerable population.

## Materials and methods

### Study design and patient selection

This retrospective cohort study was conducted at a tertiary care medical center. The study protocol was approved by the Institutional Review Board, and informed consent requirement was waived due to the retrospective nature of data collection. Consecutive elderly patients (aged ≥65 years) who underwent VATS for primary lung cancer from 12 November 2023 to 13 August 2025 were screened for eligibility. Patients were categorized into the CPSP group (n = 100) and the non-CPSP group (n = 100) according to whether pain persisted for at least 3 months postoperatively. Patients in both groups were drawn from the same institutional surgical service and followed identical perioperative protocols throughout the study period. Inclusion criteria comprised: (1) age ≥65 years; (2) histologically confirmed primary lung cancer; (3) elective VATS lobectomy, segmentectomy, or wedge resection; (4) complete perioperative and follow-up data availability; and (5) minimum 6-month postoperative follow-up. Exclusion criteria included conversion to thoracotomy, palliative surgery, reoperation within 6 months, metastatic disease, severe cognitive impairment, previous thoracic surgery, and incomplete data records. Based on these criteria, 200 patients were included in the final analysis.

### Chronic postsurgical pain definition and assessment

Chronic postsurgical pain was defined according to International Association for the Study of Pain (IASP) criteria as pain persisting or developing after VATS, lasting at least 3 months postoperatively, localized to the surgical area or referred to the ipsilateral chest wall, and not attributable to other causes such as cancer recurrence, infection, or other identifiable pathology. Pain assessment was performed using the Numeric Rating Scale (NRS, 0–10) at multiple time points: preoperatively, daily during hospitalization, at discharge, and at 1, 3, and 6 months postoperatively. Patients were categorized into the CPSP group if they reported NRS ≥3 at 3 months postoperatively, while those with NRS <3 or no pain were classified as the non-CPSP group.

### Data collection

Comprehensive demographic, clinical, surgical, and perioperative data were extracted from electronic medical records. Demographic variables included age, gender, body mass index (BMI), smoking history, and alcohol consumption. Comorbidities were documented including hypertension, diabetes mellitus, coronary artery disease, chronic obstructive pulmonary disease (COPD), and osteoarthritis. Preoperative chronic pain conditions, analgesic medication use, and psychological factors (anxiety or depression, evaluated via HADS when available) were recorded. Tumor-related variables included histological type, location, pathological TNM stage, and size. Surgical parameters encompassed procedure type, lymph node dissection extent, operative duration, number of ports, intraoperative blood loss, and chest tube duration. Postoperative variables included acute pain scores during the first 48 h, analgesic consumption, length of hospital stay, and complications classified by the Clavien-Dindo system.

### Statistical analysis

Statistical analyses were performed using SPSS version 26.0 and R version 4.2.0. Continuous variables were expressed as mean ± standard deviation (SD) or median (interquartile range). Categorical variables were presented as frequencies and percentages. Between-group comparisons used Student’s t-test or Mann-Whitney U test for continuous variables and chi-square or Fisher’s exact test for categorical variables. Univariate logistic regression was performed to screen potential risk factors (p < 0.10 threshold). Significant variables were entered into multivariate logistic regression using backward stepwise elimination (entry p < 0.05; removal p > 0.10). Multicollinearity was assessed using variance inflation factor (VIF<5). An early warning scoring system was developed based on regression β coefficients, and ROC curve analysis was used to evaluate discriminatory performance. Optimal cut-off was determined by the Youden index. Model calibration was assessed using the Hosmer-Lemeshow test. Internal validation used bootstrap resampling with 1,000 iterations.

## Results

### Patient characteristics and CPSP incidence

A total of 200 elderly patients met inclusion criteria. The cohort comprised 105 females (52.5%) and 95 males (47.5%), with a mean age of 71.4 ± 3.8 years (range, 65‐81 years). The overall incidence of CPSP at 3 months postoperatively was 50.0% (100/200). The mean age was significantly higher in the CPSP group compared to the non-CPSP group (72.4 ± 3.7 vs. 70.3 ± 3.6 years, p < 0.001). Female gender was more prevalent in the CPSP group (62.0% vs. 43.0%, p = 0.007). BMI did not differ significantly between groups (23.4 ± 3.2 vs. 23.8 ± 2.9 kg/m^2^, p = 0.352). Preoperative chronic pain conditions were significantly more prevalent in the CPSP group (32.0% vs. 9.0%, p < 0.001), as were documented anxiety or depression diagnoses (28.0% vs. 12.0%, p = 0.004). Baseline demographic and clinical characteristics are detailed in Table 1.

**TABLE 1:** Baseline demographic and clinical characteristics.

Variable	CPSP group (n = 100)	Non-CPSP group (n = 100)	P value
Age (years), mean ± SD	72.4 ± 3.7	70.3 ± 3.6	<0.001
Age ≥75 years, n (%)	26 (26.0)	15 (15.0)	<0.001
Female gender, n (%)	62 (62.0)	43 (43.0)	0.007
BMI (kg/m^2^), mean ± SD	23.4 ± 3.2	23.8 ± 2.9	0.352
BMI >25 kg/m^2^, n (%)	36 (36.0)	28 (28.0)	0.201
Smoking history, n (%)	42 (42.0)	38 (38.0)	0.552
Hypertension, n (%)	58 (58.0)	54 (54.0)	0.556
Diabetes mellitus, n (%)	26 (26.0)	22 (22.0)	0.494
COPD, n (%)	18 (18.0)	16 (16.0)	0.696
Preoperative chronic pain, n (%)	32 (32.0)	9 (9.0)	<0.001
Anxiety/Depression, n (%)	28 (28.0)	12 (12.0)	0.004
Adenocarcinoma, n (%)	72 (72.0)	76 (76.0)	0.508
Upper lobe tumor, n (%)	64 (64.0)	62 (62.0)	0.770
Stage IA–IB, n (%)	58 (58.0)	64 (64.0)	0.384

CPSP: chronic postsurgical pain; SD: standard deviation; BMI: body mass index; COPD: chronic obstructive pulmonary disease. Data expressed as mean ± SD or n (%). P values by Student’s t-test or chi-square test.

### Surgical and perioperative factors

Lobectomy was the most common procedure in both groups (58.0% vs. 52.0%, p = 0.388). Extensive surgical resection requiring systematic mediastinal lymph node dissection was significantly more frequent in the CPSP group (68.0% vs. 42.0%, p < 0.001). Mean operative duration was significantly longer in CPSP patients (182.4 ± 48.6 vs. 165.2 ± 42.8 min, p = 0.006). Intraoperative blood loss >50 mL occurred in 48.0% of CPSP patients versus 26.0% of non-CPSP patients (p = 0.001). Postoperative chest tube duration was prolonged in the CPSP group (4.8 ± 2.1 vs. 3.9 ± 1.6 days, p = 0.001). Acute postoperative pain control was significantly worse in the CPSP group (NRS 5.2 ± 1.4 vs. 3.6 ± 1.2, p < 0.001), with 74.0% versus 32.0% having inadequate pain control (NRS ≥4, p < 0.001). Length of hospital stay was longer in CPSP patients (8.6 ± 3.2 vs. 7.2 ± 2.4 days, p < 0.001). Surgical and perioperative characteristics are summarized in Table 2.

**TABLE 2:** Surgical and perioperative characteristics.

Variable	CPSP group (n = 100)	Non-CPSP group (n = 100)	P value
Lobectomy, n (%)	58 (58.0)	52 (52.0)	0.388
Segmentectomy, n (%)	28 (28.0)	30 (30.0)	0.744
Wedge resection, n (%)	14 (14.0)	18 (18.0)	0.430
Lymph node dissection (systematic), n (%)	68 (68.0)	42 (42.0)	<0.001
Operative duration (min), mean ± SD	182.4 ± 48.6	165.2 ± 42.8	0.006
Operative duration >180 min, n (%)	54 (54.0)	34 (34.0)	0.005
Intraoperative blood loss >50 mL, n (%)	48 (48.0)	26 (26.0)	0.001
Chest tube duration (days), mean ± SD	4.8 ± 2.1	3.9 ± 1.6	0.001
Hospital stay (days), mean ± SD	8.6 ± 3.2	7.2 ± 2.4	<0.001
Acute pain NRS, mean ± SD (0–10)	5.2 ± 1.4	3.6 ± 1.2	<0.001
Inadequate acute pain control (NRS≥4), n (%)	74 (74.0)	32 (32.0)	<0.001
Postoperative complications, n (%)	24 (24.0)	16 (16.0)	0.149
Prolonged air leak, n (%)	12 (12.0)	8 (8.0)	0.338
Pneumonia, n (%)	8 (8.0)	6 (6.0)	0.571

CPSP: chronic postsurgical pain; NRS: numeric rating scale; SD: standard deviation. Inadequate acute pain control defined as mean NRS ≥4 during the first 48 postoperative hours.

### Univariate and multivariate analysis of risk factors

Univariate logistic regression identified 9 variables significantly associated with CPSP development (p < 0.10): age ≥75 years, female gender, preoperative chronic pain, anxiety/depression, tumor size >3 cm, extensive lymph node dissection, operative duration >180 min, intraoperative blood loss >50 mL, and inadequate acute pain control (Table 3). These variables were entered into multivariate analysis.

**TABLE 3:** Univariate logistic regression analysis of potential risk factors for chronic postsurgical pain.

Variable	CPSP group n (%)	Non-CPSP group n (%)	OR (95% CI) P value
Age ≥75 years	26 (26.0)	15 (15.0)	1.99 (0.98−4.04) 0.010
Female gender	62 (62.0)	43 (43.0)	2.16 (1.23−3.81) 0.008
BMI >25 kg/m^2^	36 (36.0)	28 (28.0)	1.45 (0.82–2.58) 0.201
Preoperative chronic pain	32 (32.0)	9 (9.0)	4.74 (2.15–10.44) <0.001
Anxiety/Depression	28 (28.0)	12 (12.0)	2.85 (1.35–6.01) 0.006
Smoking history	42 (42.0)	38 (38.0)	1.18 (0.67–2.09) 0.552
Tumor size >3 cm	38 (38.0)	26 (26.0)	1.74 (0.97–3.13) 0.065
Extensive lymph node dissection	68 (68.0)	42 (42.0)	2.93 (1.65–5.19) <0.001
Operative duration >180 min	54 (54.0)	34 (34.0)	2.27 (1.29–4.00) 0.005
Intraoperative blood loss >50 mL	48 (48.0)	26 (26.0)	2.62 (1.46–4.68) 0.001
Inadequate acute pain control	74 (74.0)	32 (32.0)	6.04 (3.32–10.98) <0.001
Postoperative complications	24 (24.0)	16 (16.0)	1.65 (0.83–3.30) 0.149

OR: odds ratio; CI: confidence interval; BMI: body mass index. Variables with P < 0.10 were included in multivariate analysis.

Multivariate logistic regression with backward stepwise elimination identified seven independent risk factors for CPSP development (Table 4): advanced age ≥75 years (OR = 3.24, 95% CI: 1.78–5.89, p < 0.001), female gender (OR = 2.16, 95% CI: 1.24–3.76, p = 0.007), preoperative chronic pain (OR = 4.52, 95% CI: 2.15–9.51, p < 0.001), anxiety or depression (OR = 2.87, 95% CI: 1.52–5.42, p = 0.001), extensive surgical resection requiring lymph node dissection (OR = 3.08, 95% CI: 1.65–5.75, p < 0.001), intraoperative blood loss >50 mL (OR = 2.45, 95% CI: 1.32–4.54, p = 0.005), and inadequate acute postoperative pain control (OR = 3.76, 95% CI: 1.98–7.14, p < 0.001). The Hosmer-Lemeshow test indicated good model fit (χ^2^ = 6.842, p = 0.553), and all VIF values were <2.0, indicating absence of significant multicollinearity.

**TABLE 4:** Multivariate logistic regression analysis of independent risk factors for chronic postsurgical pain.

Risk factor	β coefficient	OR (95% CI)	P value
Age ≥75 years	1.175	3.24 (1.78–5.89)	<0.001
Female gender	0.770	2.16 (1.24–3.76)	0.007
Preoperative chronic pain	1.508	4.52 (2.15–9.51)	<0.001
Anxiety/Depression	1.054	2.87 (1.52–5.42)	0.001
Extensive lymph node dissection	1.125	3.08 (1.65–5.75)	<0.001
Intraoperative blood loss >50 mL	0.896	2.45 (1.32–4.54)	0.005
Inadequate acute pain control	1.325	3.76 (1.98–7.14)	<0.001

OR: odds ratio; CI: confidence interval. Hosmer-Lemeshow goodness-of-fit: χ^2^ = 6.842, P = 0.553. All variance inflation factor values <2.0.

### Development of early warning scoring system

Based on the β coefficients from multivariate analysis, an early warning scoring system was developed with integer points assigned proportionally to each risk factor’s β coefficient (Table 5). Specifically, factors with β ≥ 1.15 were assigned 3 points (preoperative chronic pain β = 1.508; inadequate acute pain control β = 1.325; age ≥75 years β = 1.175), and factors with β between 0.7 and 1.14 were assigned 2 points (extensive lymph node dissection β = 1.125; anxiety/depression β = 1.054; intraoperative blood loss >50 mL β = 0.896; female gender β = 0.770), yielding a total possible score of 0–17 points. The system comprised: age ≥75 years (3 points), inadequate acute pain control (3 points), preoperative chronic pain (3 points), extensive lymph node dissection (2 points), anxiety/depression (2 points), intraoperative blood loss >50 mL (2 points), and female gender (2 points).

**TABLE 5:** Early warning scoring system for chronic postsurgical pain risk after VATS in elderly patients.

Risk factor	Points assigned	Rationale (OR from multivariate)
Age ≥75 years	3	OR = 3.24
Inadequate acute pain control	3	OR = 3.76
Preoperative chronic pain	3	OR = 4.52
Extensive lymph node dissection	2	OR = 3.08
Anxiety/Depression	2	OR = 2.87
Intraoperative blood loss >50 mL	2	OR = 2.45
Female gender	2	OR = 2.16
Total possible score	0–17	—

Points assigned proportionally to the β coefficient of each independent risk factor from multivariate logistic regression. VATS: video-assisted thoracoscopic surgery.

Risk stratification categories were established based on score distribution and CPSP probability: low risk (0–4 points), moderate risk (5–8 points), and high risk (≥9 points). Among low-risk patients (n = 48), only 4 (8.3%) developed CPSP. In the moderate-risk category (n = 88), 42 patients (47.7%) developed CPSP. In the high-risk category (n = 64), 54 patients (84.4%) developed CPSP. The proportion developing CPSP increased significantly across risk categories (χ^2^ for trend = 98.6, p < 0.001; Table 6).

**TABLE 6:** Risk stratification categories and CPSP probability of the early warning scoring system.

Risk category	Score range	n (patients)	CPSP cases n (%)	CPSP probability
Low risk	0–4	48	4 (8.3%)	8.3%
Moderate risk	5–8	88	42 (47.7%)	47.7%
High risk	≥9	64	54 (84.4%)	84.4%
Total	0–17	200	100 (50.0%)	50.0%

AUC: area under the ROC curve; CI: confidence interval; PPV: positive predictive value; NPV: negative predictive value; CPSP: chronic postsurgical pain. Overall model performance: AUC = 0.847 (95% CI: 0.794–0.900); sensitivity = 82.0%; specificity = 78.0%; PPV = 79.6%; NPV = 80.5%; accuracy = 80.0% at optimal cut-off score = 7.

### Predictive performance and validation

**FIGURE 1:**
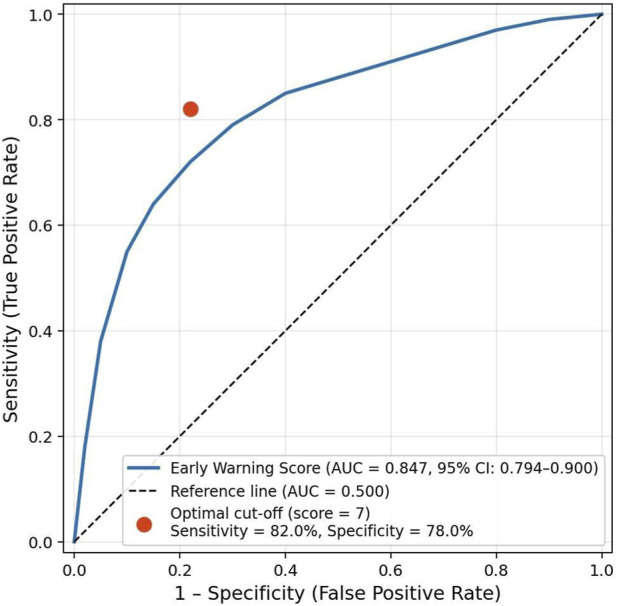
Receiver operating characteristic (ROC) curve of the early warning scoring system for predicting chronic postsurgical pain (CPSP) after video-assisted thoracoscopic surgery (VATS) in elderly lung cancer patients. The area under the curve (AUC) was 0.847 (95% CI: 0.794–0.900, P < 0.001). The red dot indicates the optimal cut-off value of 7 points (Youden index), yielding sensitivity 82.0%, specificity 78.0%, and accuracy 80.0%. The dashed diagonal represents the reference line (AUC = 0.500).

**FIGURE 2:**
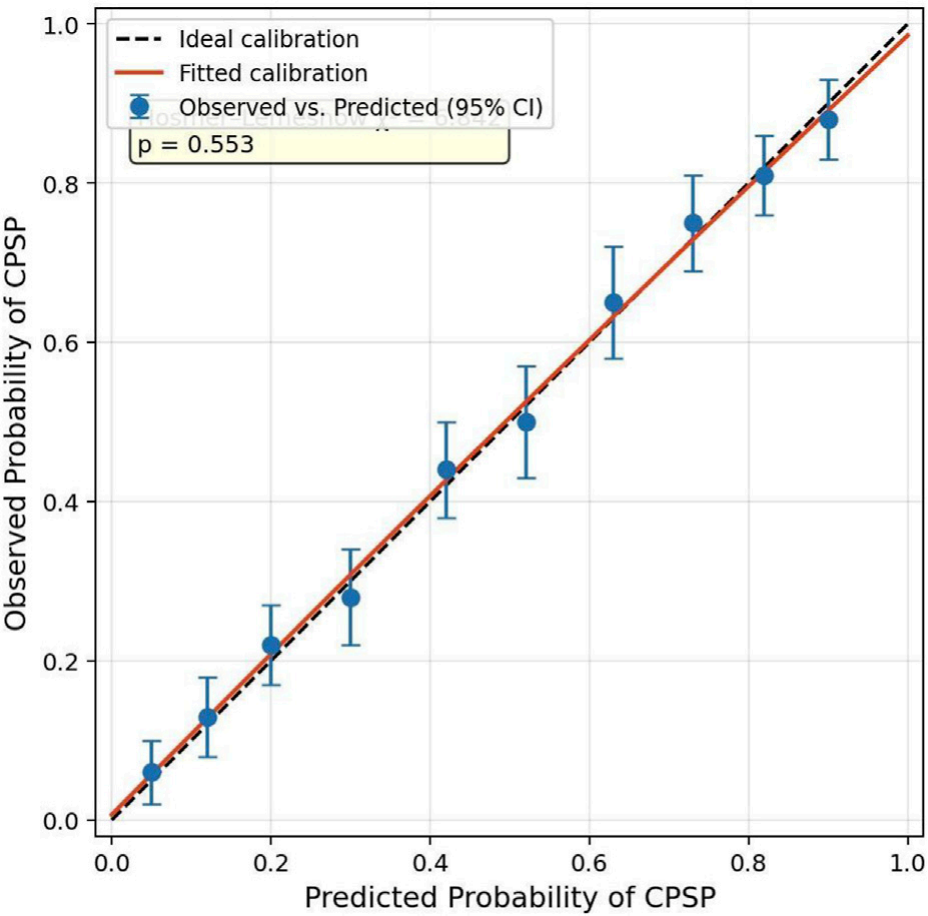
Calibration curve demonstrating agreement between predicted probabilities and observed frequencies of CPSP across the scoring range. Data points represent observed CPSP probability within each score decile (error bars = 95% CI). The dashed diagonal represents ideal calibration. The red fitted curve closely approximates the ideal line, confirming good calibration (Hosmer-Lemeshow χ^2^ = 6.842, P = 0.553).

ROC curve analysis demonstrated good discriminatory performance with AUC of 0.847 (95% CI: 0.794–0.900, p < 0.001) (Figure 1). At the optimal cut-off value of 7 points (determined by Youden index), the scoring system achieved sensitivity of 82.0%, specificity of 78.0%, positive predictive value of 79.6%, negative predictive value of 80.5%, and overall accuracy of 80.0%. Calibration curve analysis showed good agreement between predicted and observed CPSP probabilities across the full risk spectrum (Figure 2), confirmed by Hosmer-Lemeshow test (χ^2^ = 6.842, p = 0.553). Internal validation using bootstrap resampling (1,000 iterations) demonstrated model stability with mean AUC of 0.842 (95% CI: 0.788–0.896). Sensitivity analysis excluding patients with postoperative complications yielded similar results (AUC = 0.839, 95% CI: 0.783–0.895). It should be noted that these performance metrics are derived from internal validation only; the AUC of 0.847 should be interpreted with caution and should not be considered a definitive estimate of generalizability pending prospective external multicenter validation.

### Clinical application scenarios

To illustrate practical application, consider three representative patients. Patient A: a 68-year-old male without preoperative pain or psychological comorbidities, undergoing wedge resection with minimal blood loss and adequate postoperative analgesia, scores 0 points (low risk, predicted CPSP probability 8.3%). Patient B: a 72-year-old female with mild anxiety but no chronic pain, undergoing lobectomy with lymph node dissection and moderate blood loss, scores 6 points (moderate risk, predicted CPSP probability 47.7%). Patient C: a 78-year-old female with preoperative chronic back pain and depression, undergoing extensive resection with significant blood loss and suboptimal acute pain control, scores 17 points (high risk, predicted CPSP probability 84.4%). These scenarios demonstrate the scoring system’s utility in identifying patients requiring enhanced preventive strategies.

## Discussion

This study successfully identified seven independent clinical determinants of CPSP following VATS in elderly lung cancer patients and established a validated early warning scoring system with good predictive performance. The 50.0% incidence of CPSP observed in our elderly cohort aligns with the higher end of reported ranges in the literature, reflecting the unique vulnerability of this population to pain chronification and underscoring the clinical importance of systematic preoperative risk stratification and targeted preventive strategies.

Advanced age emerged as a potent independent risk factor, with patients ≥75 years demonstrating 3.24-fold increased CPSP risk compared to younger elderly individuals. This finding contrasts with some previous studies suggesting protective effects of advanced age but aligns with recent evidence highlighting age-related vulnerability factors including compromised endogenous pain modulation, reduced physiological reserves, increased inflammatory responses, and impaired tissue healing. The threshold of 75 years may represent a critical inflection point where cumulative age-related changes substantially impact pain chronification processes. These findings underscore the necessity of age-stratified approaches in elderly surgical populations.

Female gender’s association with increased CPSP risk (OR = 2.16) is consistent with extensive literature documenting sex differences in pain perception, processing, and chronification (Fillingim et al., 2009; Mogil, 2012). Potential mechanisms include hormonal influences on pain pathways, sex-specific differences in endogenous opioid systems, higher anxiety prevalence, and biological differences in inflammatory responses and neuroplasticity. Recognition of sex-specific risk enables implementation of gender-sensitive pain management approaches including dose adjustments of analgesics, enhanced psychological support, and consideration of hormonal status in treatment planning.

Preoperative chronic pain demonstrated the strongest association with CPSP development (OR = 4.52), highlighting the critical importance of comprehensive preoperative pain assessment. Existing chronic pain conditions indicate altered central pain processing, central sensitization, and compromised descending inhibitory pathways that predispose to pain chronification after surgical nociceptive input. This finding emphasizes the necessity of preoperative optimization in patients with chronic pain, including maximizing preoperative pain control, consideration of preventive interventions such as ketamine or gabapentinoids, and establishment of realistic postoperative expectations through patient education.

Anxiety and depression’s role as independent risk factors (OR = 2.87) underscores the biopsychosocial nature of CPSP. Psychological distress influences pain perception through multiple mechanisms including attentional biases toward pain-related stimuli, catastrophic thinking patterns, reduced coping capacity, and neurobiological alterations involving hypothalamic-pituitary-adrenal axis dysregulation and altered neurotransmitter systems. Integration of psychological screening and perioperative psychological interventions including cognitive-behavioral therapy, mindfulness-based approaches, and appropriate psychopharmacological management may significantly impact CPSP outcomes.

Extensive surgical resection requiring lymph node dissection increased CPSP risk 3.08-fold, reflecting the impact of surgical trauma extent on pain chronification. Lymph node dissection involves greater tissue disruption, longer operative duration, increased inflammatory responses, and higher probability of intercostal nerve injury, which may contribute to the neuropathic component of persistent postsurgical pain (Haroutiunian et al., 2013). While oncological considerations must guide surgical decision-making, awareness of associated CPSP risk can inform surgical planning including meticulous technique to minimize nerve trauma and implementation of enhanced multimodal analgesia protocols for extensive procedures.

Intraoperative blood loss >50 mL emerged as a modifiable surgical factor associated with 2.45-fold increased CPSP risk. Although VATS is generally associated with minimal blood loss, increased bleeding reflects surgical complexity, tissue trauma extent, and inflammatory response magnitude. Optimization of surgical technique, meticulous hemostasis, and enhanced recovery protocols targeting inflammatory responses may mitigate this risk factor.

Inadequate acute postoperative pain control demonstrated strong association with CPSP (OR = 3.76), providing compelling evidence for the critical importance of aggressive acute pain management. Poorly controlled acute pain triggers peripheral and central sensitization processes that facilitate transition to chronic pain through neuroplastic changes in peripheral nociceptors, spinal cord neurons, and supraspinal pain processing centers. This finding supports implementation of proactive multimodal analgesia protocols including scheduled non-opioid analgesics, regional anesthetic techniques, and individualized opioid therapy.

The developed early warning scoring system addresses a meaningful gap in perioperative medicine by providing a practical, clinically derived tool for CPSP risk stratification in elderly VATS patients. The scoring system demonstrated good discriminatory performance (AUC = 0.847) with clinically meaningful differentiation across risk categories. The 0–17 point scoring range facilitates rapid bedside calculation, while the three-tier risk stratification provides clear decision support for implementing risk-appropriate interventions. However, as this performance was established in a single-center cohort with internal bootstrap validation only, the AUC estimate should be interpreted with appropriate caution. Prospective external multicenter validation is required before the scoring system can be recommended for routine clinical deployment.

Clinical implementation enables several strategic interventions. Low-risk patients (0–4 points) can receive standard perioperative care with confidence in favorable outcomes. Moderate-risk patients (5–8 points) warrant enhanced monitoring and proactive multimodal analgesia including regional techniques such as thoracic epidural or paravertebral blocks, scheduled adjuvant medications (gabapentinoids, NSAIDs), and early physiotherapy. High-risk patients (≥9 points) require intensive preventive strategies including preoperative psychological intervention, aggressive multimodal analgesia with regional techniques, preventive medications (ketamine, gabapentin), intensive acute pain management with dedicated pain service involvement, extended postoperative monitoring, and structured transition to chronic pain management if needed.

Several limitations warrant consideration. First, the retrospective single-center design limits generalizability and may introduce selection bias. Second, the model relies solely on bootstrap internal validation; the AUC of 0.847 should not be interpreted as a validated generalizability estimate, and prospective multicenter external validation is an essential prerequisite to clinical implementation. Second, some potentially relevant factors including genetic polymorphisms, detailed medication histories, and quantitative sensory testing parameters were not assessed. Fourth, the heterogeneous ascertainment of anxiety and depression (formal HADS screening versus medical record diagnosis) may introduce information bias that affects the precision of this variable’s effect estimate. Fifth, the 3-month time point for CPSP assessment may not capture all cases, as some patients develop pain later. Sixth, the study focused on elderly patients, limiting applicability to younger populations. Finally, the relatively modest sample size necessitates external validation in larger cohorts to confirm model robustness.

## Conclusion

This study identified seven independent clinical predictors of CPSP following VATS in elderly lung cancer patients: advanced age ≥75 years, female gender, preoperative chronic pain, anxiety/depression, extensive lymph node dissection, intraoperative blood loss >50 mL, and inadequate acute postoperative pain control. The developed early warning scoring system demonstrated good predictive performance with AUC of 0.847, enabling preoperative risk stratification into low-risk (8.3% probability), moderate-risk (47.7%), and high-risk (84.4%) categories. This practical, clinically derived tool provides actionable decision support for implementing individualized perioperative pain management strategies, potentially improving long-term pain outcomes and quality of life in elderly lung cancer survivors. Prospective external multicenter validation is required before routine clinical implementation of this scoring system.

## Data Availability

The datasets generated and analyzed during the current study are not publicly available due to patient privacy and ethical restrictions. De-identified data may be made available by the corresponding author upon reasonable request and subject to approval by the institutional ethics committee.
